# Influence of pain duration and severity on oral health-related quality of life and patient satisfaction during adult treatment with clear aligners

**DOI:** 10.1186/s40510-024-00514-6

**Published:** 2024-04-29

**Authors:** Waleska Caldas, Fabiana Aparecida Bonin, Camila Pereira Vianna, Roberto Hideo Shimizu, Larissa Carvalho Trojan

**Affiliations:** 1 Ilapeo College, R. Jacarezinho, 656, Curitiba, PR 80710-150 Brazil; 2 Private Practice, Curitiba, Brazil; 3 Neodent, Curitiba, Brazil; 4 Straumann Group, Irvine, US

**Keywords:** Quality of life, Patient satisfaction, Surveys and questionnaires, Orthodontic appliances, Oral health

## Abstract

**Background:**

This study aims to compare the impact of pain on quality of life and patient satisfaction during treatment with aligners.

**Methods:**

Ninety-four subjects in active treatment were invited to answer self-reported questions concerning pain severity and duration, occurrence of other signs and symptoms, and level of satisfaction with their treatment. Also, the OHIP-14 questionnaire was applied to assess patients’ OHRQoL.

**Results:**

Ninety-four patients (49 women and 45 men) answered the survey. Pain duration of 1–3 days was reported by 69.1% of patients (n = 60). For those who reported pain (n = 84), it was considered mild severity by 42.9% and moderate by 52.4%. Almost sixty-four percent of the sample were very satisfied with the aligner’s aesthetics (n = 60) and forty-nine percent were satisfied with treatment in general (n = 46). Mean OHIP-14 score was 3.36 ± 2.54. OHRQoL was significantly associated with pain severity, whereas patients who reported having experienced moderate pain presented a significantly higher mean OHIP-14 score than those who reported having experienced mild pain (3.92 ± 1.93 and 2.69 ± 2.83, respectively; *p* = 0.036). The “psychological discomfort” OHIP-14’s domain was the most influenced by the level of pain.

**Conclusion:**

Pain severity significantly influenced OHRQoL, in adult patients under treatment with clear aligners. However, high levels of patient satisfaction were reported, regardless of pain duration or severity.

## Introduction

Modern orthodontics is striving to offer patients a comfortable and successful treatment experience. However, it is well known that orthodontic treatment is usually associated with several adverse effects, such as pain, anxiety, and decreased oral health-related quality of life (OHRQoL) [1].

One of the most commonly reported impacts of orthodontic therapy are difficulties during function (speaking and chewing) and impairment of smile esthetics [2]. These aspects can negatively impact the level of patient satisfaction during treatment, especially during the initial stages, when the pain is expected to be at higher levels [3–5]. In addition to oral health-related quality of life, patient satisfaction may be influenced by several other factors such as gender, age, treatment duration, and dentofacial improvement [6, 7]. In this way, self-reported questionnaires have been used to measure patients perception of the impact of oral conditions as well as different treatment modalities on their well-being, representing the short version of the oral health impact profile questionnaire (OHIP-14), one of the most widely applied in the literature [2, 8, 9].

Health-related quality-of-life measures have become a popular way of assessing the impacts of a treatment on a patient's well-being. One of the factors within orthodontic therapy that presents a strong influence on a patient's wellness is the amount of pain experienced, as well as how much pain or the presence of the appliance itself may impact daily activities, such as chewing and speaking [10].

Patient satisfaction during orthodontic treatment with clear aligners is essential, as it is closely related to the level of compliance and, consequently, to the quality of the results obtained [11]. Therefore, the need to further understand what an adult patient should expect during treatment with clear aligners in terms of symptoms and discomfort, as well as how these may influence treatment satisfaction, seems to be an important factor in determining the efficacy of therapy.

Clear aligner treatment has raised the interest of adult patients, who present high demands on comfort and aesthetics of the appliances [12]. Orthodontists, in turn, seek therapies that involve high levels of patient satisfaction, as individuals who are unsatisfied with treatment may even discontinue therapy [13].

Health-related quality-of-life measures have become a popular way of assessing the impacts of a treatment on a patient's well-being. One of the factors within orthodontic therapy that presents a strong influence on a patient's wellness is the amount of pain experienced, as well as how much pain or the presence of the appliance itself may impact daily activities, such as chewing and speaking [10].

Pain after orthodontic appointments is mainly related to two phenomena: immediate pain, due to periodontal compression, and delayed pain, related to the inflammatory response caused by tooth movement. The latter usually starts 1 day after appliance change or activation and may last for 1–7 days [14].

This study aims to compare the impact of pain on quality of life and patient satisfaction during treatment with aligners. The study hypothesis is that patients with longer duration and greater severity of pain will report significantly worse levels of patient satisfaction and OHRQoL.

## Materials and methods

The present cross-sectional study was approved by the Research Ethics Committee of Curitiba University Center (Curitiba, Brazil; protocol number 08697018.5.0000.8387). The sample comprised ninety-four adult patients (the patients were over 18 years old), under active treatment with aligners (ClearCorrect®) between April 2020 and June 2022, at a private practice (Curitiba, Brazil), by a single orthodontist (F.B.) who provided written consent to participate in this questionnaire study. Exclusion criteria were unsuitable oral (presenting caries or periodontal disease) or general health (presenting systemic diseases, cognitive disorders, chronic medical conditions, or were considered vulnerable in any other way); need of orthodontic-surgical treatment.

After analyzing a database from a dental clinic and based on the study criteria, 186 patients who were being treated with aligners were selected. An online electronic questionnaire was sent via e-mail.

All patients were instructed to wear the aligners 22 h daily—removing them just to eat and to perform oral hygiene—and followed a 2-week aligner change protocol.

This survey was performed based on an online electronic questionnaire, which was developed based on an extensive literature review to assess the study parameters. Upon login, each participant was assigned to a consecutive number and was thus irreversibly anonymized. The online questionnaire (“Appendix 2”) consisted of demographic data of the subject such as age, gender, and 8 items arranged in subsections of multiple choice questions according to the following topics: 1. Treatment phase (1–3 months; 3–6 months; more than 6 months); 2. Pain duration (no pain; 1–3 days; 3–5 days; more than 5 days); 3. Pain severity (mild; moderate; severe); 4. Other signs or symptoms (gingival pressure; tongue irritation; unpleasant taste or smell; gingival bleeding; local edema; mouth opening difficulty; nausea); 5. Satisfaction with aligners’ aesthetics (very satisfied; satisfied; neither satisfied nor unsatisfied; unsatisfied); 6. Satisfaction with treatment results obtained so far (very satisfied; satisfied; neither satisfied nor unsatisfied; unsatisfied); 7. Previous conventional orthodontic treatment (yes; no); 8. If yes. Greater satisfaction with clear aligners? (yes; no; I don’t know).

Also, to assess OHRQoL, the weighted OHIP-14 questionnaire was added to the digital questionnaire, which comprised 14 questions to evaluate the domains: functional limitation, physical pain, psychological discomfort, physical, psychological, and social disability as well as handicap. The items are rated on a 5-point scale, ranging from 0 (never) to 4 (always), and the final score is the sum of all individual items, multiplied by each question weight. Accordingly, the OHIP-14 scores range from 0 to 56, where the higher scores indicated poor quality of life-related to oral health. This study used the validated version for Brazilian Portuguese, which has shown good psychometrics (intraclass coefficient = 0.87 and Cronbach’s alpha = 0.91) [15, 16].

### Statistical analyses

Descriptive summary statistics were computed for all data collected by the questionnaire. Quantitative parameters were described by means and standard deviations, and frequencies were given for the qualitative variables.

The Shapiro–Wilk test was used to verify the normal distribution of the sample. The normality results indicated the presence of variables that follow a normal distribution (*p* > 0.05). In addition, to reinforce the robustness of this analysis, the normality graphs were observed, confirming that these variables follow a normal distribution.

Intergroup comparisons considering “sex” and “treatment phase” for pain duration and severity were performed using Fisher's exact tests. The influence of the parameter’s “sex”, “treatment phase”, “pain duration” and “pain severity” on the OHIP-14 scores and probability to recommend treatment to others was verified by *t* tests and One-way ANOVA test, and on the level of treatment satisfaction with Fisher's exact tests.

All analyses were performed using Statistica Software (version 10, Statsoft, Tulsa, OK, USA). Results were regarded as significant at *p* < 0.05.

## Results

Ninety-four patients (49 women and 45 men), with a mean age of 33.9 ± 6.3 years (range 21–54 years) have provided written consent to participate and answered the survey. Of the 186 patients who received the e-mail with the online electronic questionnaire, only 94 responded (50.6%).

The main reason for seeking orthodontic treatment was to improve tooth alignment for 75 patients (79.8%). Sixty-five patients (69.1%) had been undergoing treatment for more than 6 months and 58 patients reported wearing the aligners for 16–22 h a day (61.7%). Pain duration of 1 and 3 days after the last aligner change was reported by 65 patients (69.1%). For those who reported pain (n = 84), it was considered mild by 42.9% (n = 36) and moderate by 52.4% (n = 44). About other symptoms, the most frequently reported were gingival pressure for 68.1% of patients (n = 64) and 34.0% reported tongue irritation (n = 32; Table 4—“Appendix 1”).

Regarding the aesthetics of the aligner, 60 patients (63.8%) reported being very satisfied with the aesthetics of the aligner, and 46 (48.9%) with the treatment results obtained so far. Previous treatment with fixed appliances was reported by 45.7% of individuals (n = 51) and of these, 39 patients said they were more satisfied with treatment with clear aligners (“Appendix 1”—Table 4).

In this sample, 93.07% of the patients had no need for canine/molar relationship correction, but 4.95% had a Class II canine/molar relationship and 1.98% had a Class III canine/molar relationship to be corrected. Regarding the overbite correction, 5.94% were submitted to deep bite correction and 9.9% had open bite correction during aligner treatment.

**Fig. 1:**
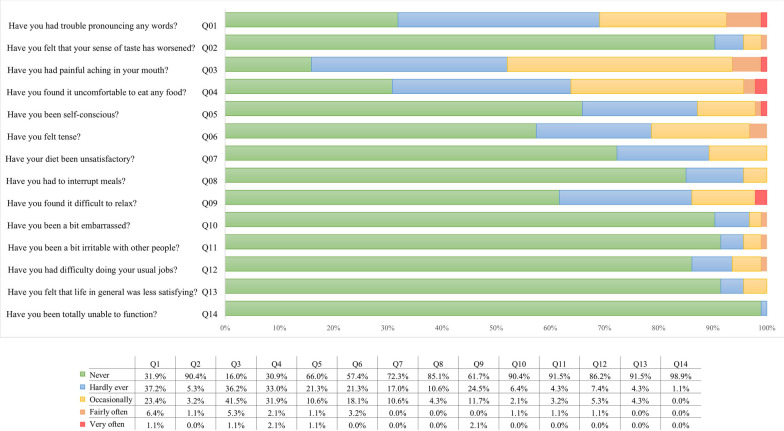
Frequency chart for OHIP-14 Questions (N = 139)

For the impact of oral conditions/treatment on patients’ quality of life, most patients reported "never" or "hardly ever" experiencing the impacts assessed by the questionnaire, resulting in a mean OHIP-14 score of 3.36 ± 2.54 (Fig. 1, Table 4).

When the influence of sex and treatment phase on pain duration and severity was verified, no statistically significant association was observed (*p* > 0.05; Table 1). Regarding the duration of pain, 34 patients reported feeling pain for 1–3 days and 27 patients reported moderate pain severity after the last aligner change, regardless of gender and treatment phase.

**Table 1:** Intergroup comparison for Pain Duration and Severity (Fisher´s Exact test)

Goups	Variables								
	Pain duration					Pain severity			
	No pain (%)	1–3 days (%)	3–5 days (%)	> 5 days (%)	*p*	Mild (%)	Moderate (%)	Severe (%)	*p*
Sex									
Female (n = 49)	10.20	69.39	16.33	4.08	0.999	38.64	54.55	6.82	0.622
Male (n = 45)	11.11	68.89	17.78	2.22		47.50	50.00	2.50	
Treatment phase									
3–6 months (n = 26)	3.85	80.77	11.54	3.85	0.534	48.00	52.00	0.00	0.484
> 6 months (n = 65)	10.77	66.15	20.00	3.08		39.66	53.45	6.90	

3 patients were in group “< 3 months”, thus, this group was not included in the intergroup analysis. However, only 1 patient reported feeling pain for 1 to 3 days, of mild severity, after last aligner change

Regarding possible influencing factors on OHIP-14 score, no statistically significant differences were observed between groups for sex, treatment phase, and pain duration (*p* > 0.05; Table 2), statistical comparison with the 1–3 months treatment phase (n = 3) and pain duration > 5 days (n = 3) groups was not possible due to insufficient sample size. On the other hand, intergroup comparison regarding pain severity showed that patients who have reported feeling moderate pain presented a significantly higher mean OHIP-14 score than those who have reported pain of mild severity (3.92 ± 1.93 and 2.69 ± 2.83, respectively; *p* = 0.036; Table 2) Statistical comparison with “severe” group was not possible due to insufficient sample (n = 4), however, this group of patients presented a higher mean OHIP-14 score than the other two groups (5.32 ± 5.06). The level of patient satisfaction with treatment and the probability they would recommend treatment to others were not significantly influenced by any of the parameters (*p* > 0.05; Table 2).

**Table 2:** Intergroup comparison for OHIP-14 score, probability to recommend treatment to others, and satisfaction with treatment results (one-way ANOVA and Fisher’s exact test)

Goups	Variables											
	N	OHIP-14			Probability to recommend treatment			Satisfaction with treatment				
		Mean	SD	*p*	Mean	SD	*p*	Unsatisfied (%)	Neither satisfied. Nor unsatisfied (%)	Satisfied (%)	Very satisfied (%)	*p*
Sex												
Female	49	3.09	2.12	0.296^a^	9.45	1.02	0.497^a^	2.04	6.12	46.94	44.90	0.660^c^
Male	45	3.64	2.93		9.58	0.78		0.00	2.22	44.44	53.33	
Treatment phase												
1–3 months	3	2.47	1.33	0.592^b^	10.00	0.00	0.275^b^	0.00	0.00	66.67	33.33	0.384^c^
3–6 months	26	3.04	2.30		9.69	0.74		0.00	11.54	42.31	46.15	
> 6 months	65	3.53	2.67		9.42	0.98		1.54	1.54	46.15	50.77	
Pain duration												
No pain	10	2.50	1.80	0.380^b^	9.60	0.70	0.786^b^	0.00	0.00	60.00	40.00	0.542^c^
1–3 days	65	3.26	2.46		9.49	0.92		1.54	4.62	43.08	50.77	
3–5 days	16	4.18	3.16		9.44	1.09		0.00	6.25	50.00	43.75	
> 5 days	3	3.96	2.66		10.00	0.00		0.00	0.00	33.33	66.67	
Pain severity												
Mild	36	2.69^A^	2.83	0.036*^b^	9.67	0.68	0.360^b^	0.00	2.78	38.89	58.33	0.335^c^
Moderate	44	3.92^B^	1.93		9.39	1.06		2.27	6.82	50.00	40.91	
Severe	4	5.32	5.06		9.25	1.50		0.00	0.00	25.00	75.00	

*SD* standard deviation

^a^T test *t*

^b^One-way ANOVA

^c^Fisher’s exact test

*Statistically significant for *p* < 0.05. Different letters indicate statistically significant differences. The comparison with the “severe” group was not possible because it did not present enough cases

When the domains of the OHIP-14 questionnaire were evaluated separately, it was observed that patients who reported experiencing moderate pain presented significantly higher scores regarding psychological discomfort (feeling self-conscious or tense) than those who reported experiencing mild pain (*p* = 002, respectively; Table 3).

**Table 3:** Pain severity intergroup comparison for OHIP-14 domains (One-way ANOVA)

OHIP-14 domain	Pain severity						
	Mild (n = 36)		Moderate (n = 44)		Severe (n = 4)		*p*
	Mean	SD	Mean	SD	Mean	SD	
Functional limitation	0.54	0.48	0.76	0.66	0.89	0.49	0.175
Physical pain	1.07	0.84	1.43	0.67	1.18	0.63	0.105
Psychological discomfort	0.33^A^	0.56	0.70^B^	0.60	0.98	0.86	0.011*
Physical disability	0.22	0.44	0.38	0.50	0.38	0.76	0.353
Psychological disability	0.32	0.70	0.42	0.43	0.90	1.15	0.187
Social disability	0.11	0.30	0.18	0.44	0.60	1.19	0.113
Handicap	0.10	0.30	0.05	0.21	0.40	0.80	0.085

*SD* standard deviation

*Statistically significant for *p* < 0.05. Different letters indicate statistically significant difference. The comparison with the “severe” group was not possible because it does not present a sufficient number of cases

## Discussion

The study hypothesis was partially rejected as, although pain severity seemed to play an important role in OHRQoL, pain duration and satisfaction did not significantly influence it.

Sixty-five (69.1%) in the present study reported that after the last aligner change, they felt pain for 1–3 days, whereas 10 (10.6%) stated that they did not feel any pain. This corroborates with other studies that reported that patients had pain peaks for this same period, with a gradual decrease [17, 18].

In the present study, pain severity was considered mild by 36 patients (42.9%) of the patients and moderate by 44 (52.4%). Accordingly, studies have reported low levels of pain and discomfort during treatment with clear aligners, with most distress being related to soft tissue irritations [19]. Corroborating with this, the most cited symptoms by the study patients were gingival pressure (68.1%) and tongue irritation (34.0%). No significant differences were observed regarding pain duration or severity between different sexes or patients under different stages of treatment.

No significant differences were observed for mean OHIP-14 scores between different sexes, treatment phases, or pain duration, whereas very low scores (i.e. low impact on quality of life) were obtained for all groups evaluated for these factors. These findings corroborate a previous study that has shown that patients treated with clear aligners presented minor social and psychological impacts, even during the first week of treatment [9].

Some of the clear advantages of treatment with aligners are the possibility of better teeth hygiene, and comfort for eating, in addition to aesthetics [20]. Most patients in the present study reported that they were never or hardly ever uncomfortable speaking, eating, or presented phycological or social discomforts (Fig. 1).

When patients in the present study were grouped according to pain severity, those who described it as moderate or severe, presented a significantly higher mean OHIP-14 score—with significantly higher scores for the psychological discomfort domain—than those who reported low pain, indicating greater influence of oral conditions on the quality of life for the first. Other authors have also shown that lower levels of pain were associated with less impact on OHRQoL, and that pain or discomfort caused by orthodontic treatment were great factors of dissatisfaction [1, 5]. However, since pain caused by aligners has been demonstrated to be significantly lower than in conventional treatments, it can be expected that this treatment modality presents fewer negative impacts in terms of patients´ quality of life [21]. Accordingly, although OHRQoL was significantly influenced by pain severity, OHIP-14 scores were still on average low for all groups and patients were still satisfied or very satisfied with treatment results, as well as highly likely to recommend aligner treatment to others.

Although pain had a negative influence on patients' quality of life, and statistically significant differences were observed between groups of patients presenting different levels of pain, the observed OHIP-14 score values were low even for patients presenting moderate and severe pain. Therefore, even these reported being mostly satisfied or very satisfied with orthodontic treatment with aligners.

The intensity of the pain influenced the patients' quality of life but did not influence their satisfaction with the treatment. Although no statistically significant difference was observed in terms of satisfaction, the short duration and low severity of the pain may be related to the high level of satisfaction during all periods of treatment. There is a tendency for the rate of satisfied patients to increase as treatment progresses.

The main limitation of this study is the subjective nature of pain assessment in patients undergoing orthodontic treatment at different stages. The experience of pain is inherently variable and personal, which may make direct comparisons between participants difficult. Despite efforts to mitigate these limitations through standardized questionnaires, it is crucial to recognize that the subjectivity of pain remains challenging in interpreting the results. It is also a limitation that it was not possible to track changes in the severity and duration of pain over the course of treatment for the same patient, due to the cross-sectional nature of the study. These points are important to consider in future studies.

The questionnaire used instrument feasibility, reliability, and validity of the Brazilian version of the Oral health-related quality of life (OHRQoL) [22]. Although the questionnaire was based on a study in the literature, there is a limitation in this study in the absence of a formal verification of the validity of the questionnaire used to assess pain in orthodontic treatments with aligners [23]. Our results indicate that treatment with clear aligners, in general, did not result in long or intense periods of pain, and did not have a major impact on adult patients' daily activities, such as eating, speaking, smiling, or overall quality of life.

## Conclusion

In the present questionnaire study including adult patients under treatment with clear aligners:

Patients with greater severity of pain had worse levels of quality of life (OHRQoL). However, the level of patient satisfaction with treatment was not significantly influenced either by the duration or by the severity of pain.

OHRQoL was significantly associated with pain severity, whereas patients who reported having experienced moderate pain presented a significantly higher mean OHIP-14 score than those who reported having experienced pain of mild severity. Further analysis showed that the domain of “psychological discomfort” was the most influenced by the level of pain.

## Data Availability

The datasets used and/or analyzed during the current study are available from the corresponding author on reasonable request.

## Appendix 1

See Table 4.

**Table 4:** Descriptive summary statistics results (n = 139)

Qualitative variables	N	Frequency (%)
Sex		
Female	49	52.13
Male	45	47.87
Treatment motivation		
Teeth alignment	75	79.79
Improve mastication	2	2.13
Prevention of future gingival or oral diseases	3	3.19
Smile with confidence and make a good impression on others	9	9.57
Other	5	5.32
Treatment phase		
1–3 months	3	3.19
3–6 months	26	27.66
> 6 months	65	69.15
Daily aligner wear time		
8–16 h/day	4	4.26
16–22 h/day	58	61.70
≥ 22 h/day	32	34.04
Pain duration		
None (no pain)	10	10.64
1–3 days	65	69.15
3–5 days	16	17.02
> 5 days	3	3.19
Pain severity		
Mild	36	42.86
Moderate	44	52.38
Severe	4	4.76
Other signs/symptoms		
Gingival pressure	64	68.09
Tongue irritation	32	34.04
Unpleasant taste or smell	23	24.47
Gingival bleeding	18	19.15
Local edema	13	13.83
Mouth opening difficulty	6	6.38
Nausea	5	5.32
Satisfaction with aligners’ aesthetics		
Very satisfied	60	63.83
Satisfied	32	34.04
Neither satisfied. Nor unsatisfied	2	2.13
Satisfaction with treatment		
Very satisfied	46	48.94
Satisfied	43	45.74
Neither satisfied. Nor unsatisfied	4	4.26
Unsatisfied	1	1.06
Previous conventional orthodontic treatment		
Yes	43	45.74
No	51	54.26
If yes. Greater satisfaction with clear aligners?		
Yes	39	90.70
No	0	0.00
I don’t know	4	9.30

Quantitative variables	Mean	SD
Age (years)	33.93	6.30
OHIP-14 score	3.36	2.54
